# Molecular Responses during Plant Grafting and Its Regulation by Auxins, Cytokinins, and Gibberellins

**DOI:** 10.3390/biom9090397

**Published:** 2019-08-22

**Authors:** Anket Sharma, Bingsong Zheng

**Affiliations:** State Key Laboratory of Subtropical Silviculture, Zhejiang A&F University, Hangzhou 311300, China

**Keywords:** horticulture techniques, plant hormones, scion, stock, vascular tissue connection

## Abstract

Plant grafting is an important horticulture technique used to produce a new plant after joining rootstock and scion. This is one of the most used techniques by horticulturists to enhance the quality and production of various crops. Grafting helps in improving the health of plants, their yield, and the quality of plant products, along with the enhancement of their postharvest life. The main process responsible for successful production of grafted plants is the connection of vascular tissues. This step determines the success rate of grafts and hence needs to be studied in detail. There are many factors that regulate the connection of scion and stock, and plant hormones are of special interest for researchers in the recent times. These phytohormones act as signaling molecules and have the capability of translocation across the graft union. Plant hormones, mainly auxins, cytokinins, and gibberellins, play a major role in the regulation of various key physiological processes occurring at the grafting site. In the current review, we discuss the molecular mechanisms of graft development and the phytohormone-mediated regulation of the growth and development of graft union.

## 1. Introduction

Plant grafting is a horticulture technique in which two cut parts (rootstock and scion) of the plant are joined together, resulting in the formation of a new plant after successful connection of vascular tissues [1]. Rootstocks are usually closely related to the scion; they often belong to the same genus [2]. The success rate of any graft is dependent upon the congeniality between stock and scion. Generally, the stock and scion of the same genera form more compatible grafts in comparison to the scion and stock of different genera [3]. Grafting is extensively used to enhance the horticultural attributes and for studying the various important physiological processes like investigating the transport mechanism of different biomolecules through the junction [1]. Plant grafting is helpful in improving the health of plants, their yield, and product quality, as well as the extension of harvesting time and postharvest life [4,5,6]. Moreover, grafting enhances the resistance of plants to different biotic and abiotic stress conditions like soil and airborne pathogens, temperature, salinity, heavy metals, and water stress [4,7,8,9,10,11,12]. Weeds like parasitic plants can also be controlled using plant grafting [13]. Additionally, plant grafting enhances the uptake of nutrients from the soil and improves the competence of their utilization [14,15].

Currently, there is research on altering the architecture of crop roots so that the uptake of mineral nutrients and their utilization efficiency can be improved [16,17]. Rootstock grafting has been considered as one of the best choices to enhance the water and nutrient uptake in crop plants and improve the efficiency of their utilization [18]. The process of successful grafting involves the key steps in the following order: reunion of phloem tissue, growth of roots, and reunion of xylem tissue [19]. One of the main processes involved in the development of a graft is the connection of vascular tissues of stock and scion. Firstly, pectins are secreted by the cells at the place of graft union where they function as an adherent for both parts. Then, the process of dedifferentiation forms a callus at the grafting site followed by connection via plasmodesmata. This is followed by the division of cells around vascular tissues (pith, cortex, and cambium) accompanied by the differentiation of callus cells at the site of grafting. Finally, the connection of phloem takes place followed by the connection of xylem tissue [19,20,21]. Moreover, plant hormones like auxins, cytokinins (CKs), ethylene (ET), gibberellins (GAs), and jasmonic acid (JA) are also involved in the regulation of physiological processes taking place at the site of graft union [22,23]. Due to their highly mobile nature, these hormones have the ability to translocate in the grafted plant parts. However, auxins are the main hormones that regulate the growth and development of vascular tissues, and their crosstalk with other hormones further regulates the auxin cell signaling involved in the process of vascular tissue development. In the process of grafting, auxins are prominently involved in tissue regeneration and vascular tissue connection, both of which are key factors for the success of graft union [24]. Keeping in mind the role of plant hormones during the process of grafting, in the current review, we discuss the possible molecular mechanisms that regulate the growth of graft unions.

## 2. Molecular Responses at the Grafting Site

### 2.1. Movement of Genetic Material at the Grafting Site

#### 2.1.1. DNA (Organelle/Nuclear Genome) Movement across Graft Unions

The regulation of the developmental process at graft unions is mediated by the genetics responsible for the induction of various phenotypic changes that can be passed to the next generation [6,25]. It has been suggested that DNA is able to transfer from stock to scion via vascular uptake, and this fact was supported by random amplification of polymorphic DNA analysis, showing that DNA bands found in successfully grafted plants were identical to the DNA bands of rootstocks but were not found in those plants that contributed as the scion [25]. Stegemann and Bock [26] suggested that horizontal gene transfer (HGT) is possible during graft development. This fact was based on their experimental studies in which after grafting of genetically modified tobacco plants, they observed that plastid genes have the capability of traveling over the grafting point. The connection of vascular tissues and the formation of plasmodesmata at the grafting point provide a route for HGT. Genetic information gets exchanged the form of large pieces of DNA or whole plastid genome, but this transfer is restricted at the grafting site without any long distance movement [26]. Furthermore, Stegemann et al. [27] reported that whole chloroplast genome can move across the interspecific graft, and this movement is independent of graft orientation. Moreover, these researchers also confirmed that this chloroplast genome transfer is inherited by the next generation and is quite stable. A grafting experiment of two different species of the family Solanaceae showed that during grafting, locus-specific changes in the process of DNA methylation occurred in the grafted scions. These epigenetic changes were partially inherited by the next generation individuals obtained after self-pollination of successfully grafted plants [28]. The migration of nuclear genome also occurs between parts of the graft, which ultimately helps in the formation of stable allopolyploid and fertile plants [29]. Therefore, the grafting technique involves the transfer of genetic material (nuclear as well as plastid genome) across the joining site of the graft, and this is very important for studying the deep mechanisms of grafting along with the role of grafting in the evolution of new plant species [1].

#### 2.1.2. RNA Movement across Graft Unions

Epigenetic modification in the grafted plants is regulated by small RNA. It is evident that 24 nucleotide small RNA migrates from the scion to the stock of a successful graft and is responsible for the epigenetic alterations in the rootstock cells by modulating the process of DNA methylation [30]. Small interfering RNAs (siRNAs) play a key role in gene silencing and signals mediated by these siRNAs (which include both transgene-specific as well as endogenous siRNAs) have the ability to transmit across the site of grafting [30,31,32]. Due to their mobile ability, siRNAs originating from the stock are able to mediate gene-silencing (transcriptional) in the scion part of a successful graft. Similarly, siRNAs of the scion origin can induce gene-silencing in the stock portion, indicating the role of RNA transport across the graft unions [33]. It is believed that miRNAs have functions during the development of graft unions. At the graft site of hickory plants, cca-miR156 and cca-miR159 were found to be up-regulated during the graft development, and stimulated the process of tissue connection. Moreover, cca-miR390b was noticed to down-regulate resulting in enhanced accumulation of ATP-binding proteins [34]. Superoxide radical scavenging enzyme superoxide dismutase 4 (SOD4) helps in protection of plant cells due to oxidative stress caused during initial stages of the grafting due to tissue injury. SOD4 is a target of a miRNA named cca-miR827 [35], and this miRNA was noticed to down-regulate during early grafting stages, favoring the activation of SOD4 to scavenge harmful superoxide radicals at graft unions [34].

Messenger RNAs (mRNAs) which are mainly responsible for the coding of different proteins, are supposed to play critical roles in the process of grafting. After transportation across the graft unions, mRNAs regulate the formation of various functional proteins involved in normal growth and development of plant tissues [1]. Various recent studies have reported mRNA transfer across the graft unions. For example, movement of 138 transcripts was observed in *Arabidopsis thaliana* and *Nicotiana benthamiana* graft [36], 2006 transcripts in the graft of *Arabidopsis* ecotypes [37], almost 3000 transcripts across *Vitis vinifera* heterografts [38] and 3546 transcripts in *Cucumis sativus* and *Citrulus lanatus* grafts [39]. For mRNA movement across the graft unions, tRNA-like structure motifs are mandatory, explaining a possible mechanism behind the intercellular transport of mRNA [40,41]. This fact was supported by the results obtained after making mRNA-bearing typical structures like tRNA. These modified mRNAs were able to transport across the graft unions and possibly undergo the process of translation after long transport, resulting in the formation of functional proteins [40,41,42]. Recent study carried out on graft unions between *Nicotiana benthamiana* and *Solanum lycopersicum* revealed that mRNAs undergo degradation during their movement across the graft junction, and a few mRNAs may return back to the scion part [43]. Researchers have reported that in the grafted plants the gibberellic acid insensitive (GAI) RNA gets translocated through phloem tissue, and is responsible for the change in leaf phenotype [44]. A combined transcript of the sequence which encodes GAI and RNA tagged with GFP (green fluorescent protein) was able to translocate up to a long distance across grafting point, but no such movement was noticed for alone GFP [45].

### 2.2. Proteins at the Grafting Site

Many proteins (like chaperones) are able to bind mRNAs and these proteins are known for their roles in favoring the process of molecular transport, and have the ability to reduce the degradation of mRNAs. One of such chaperones named CmPP16 was found in *Cucurbita maxima* and noticed to assist the RNA transportation from the stock to the scion across the graft unions [46]. Another such protein, PbPTB3 in *Pyrus betlaefolia* is capable of transporting to long distances after binding with numerous mRNAs. This long-distance movement of mRNAs across the graft junction is favored by the binding of PbPTB3 to PbWoxT1 mRNA [47,48]. A cyclophilin protein SICyp1 (involved in the cell signaling pathway responsible for maintaining shoot to root ratio), which can move via phloem from the scion to the stock, favored by an enhancement in auxin response, and ultimately aid in promoting the growth of roots [49]. Studying protein trafficking during the process of plant grafting revealed that proteins from the companion cells of shoot have the ability to get transported into the stele cells of roots, and play key roles in regulating the physiology and morphology of grafted plants [50]. During the process of grafting, movement and accumulation of auxin at grafting site helps in cell wall extension, elongation and growth [51,52]. Water availability favors the elongation of cells [52]. Plasma membrane intrinsic proteins (PIPs) play key role in the process of grafting and aquaporins are involved in cellular water transport, hence regulating active cell proliferation [53,54]. At the grafting site, stimulated expression of an aquaporin named PIP1B was accompanied by enhanced water levels and cell elongation, leading to better callus formation and successful grafting [55,56]. In *Carya cathayensis*, enhancement in the expression pattern of CcPIP1;2 during grafting was found to be useful [57]. After grafting, the expression of differentially expressed proteins (DEPs) is regulated in plants. At graft unions, 341 and 369 DEPs were found to be up-regulated (above five fold up-regulated DEPs include Mortalin-like protein 28, chlorophyll a/b-binding protein and lysine histidine transporter 1-like protein) and down-regulated (above five fold up-regulated DEPs include a GRAS family transcription factor) respectively in hickory plants [58].

## 3. Plant Hormone Mediated Grafting and Its Underlying Molecular Mechanisms

### 3.1. Regulation of the Grafting by Auxins

Auxins are a class of phytohormones which are vital for regulating almost each process of plants during their life cycle [59,60,61]. The key regulatory functions are cell division, cell elongation and cell differentiation [61]. In addition to vegetative growth, auxins also regulate the reproductive biology of plants including early pollen and embryo development [59,62]. Auxins are necessary for shoot as well as root development of plants [63]. Auxins play key regulatory function in plants during the process of graft development. The polar auxin transport (PAT) maintains the levels of auxins in plant parts and is involved in the development of xylem tissue [64]. Concentration of auxins is noticed to be very asymmetric in the xylem adjacent cells (known as pericycle cells) during graft development [19]. The role of auxin transport in the process of graft union was also confirmed by experiments carried out by Matsuoka et al. [65], in which they observed that the application of triiodobenzoic acid (which causes inhibition in the auxin transport) to *Arabidopsis* seedlings resulted in the reduction of vascular cell growth at the grafting site. 

There are two main protein families involved in the regulation of PAT. They include PIN-FORMED auxin transport proteins (PINs) and proteins of ATP-binding cassette subfamily B (ABCB). These families act as efflux carriers in auxin transport [66]. Above grafting site, activation of transcripts encoding PIN1 and ABCB1 regulates the graft development [67]. Auxin influx and efflux carriers are known for their regulatory functions in regeneration and reunion of vascular tissues. Enhanced expression of genes (*CcPIN1b* and *CcLAX3*) encoding these carriers for PAT in *Carya cathayensis* was found to favor the process of grafting [68]. In *Pisum sativum*, Sauer et al. [69] noticed that PIN1 expression was enhanced at the injured stem parts, which further triggered the process of differentiation of xylem cells at that point. The regeneration and development of vascular tissues after stem injury is accompanied by change in the location of PIN1 protein [70]. The transcript levels of genes like *PIN* and *ABCB* are also altered in response to graft development [71]. In grafted *Torreya grandis* plants, regulation of AUXIN RESPONSE FACTOR (ARF) genes involved in auxin signaling is thought to further regulate the other hormonal signaling pathways and ultimately enhance the chances of graft union [72]. Up-regulation of ARF during the process of grafting further regulates various biochemical pathways promoting vascular connection between the scion and the stock [56]. The flow of auxins is controlled by PIN1 and PIN2 [73,74]. A study on *Malus* spp. by Li et al. [75] reported a considerable less growth of roots after grafting. They observed down regulation in the gene expression of *MrPIN1* (*Malus robusta* PIN1) and *MrSHR* (*M. robusta SHORT ROOT*) in roots of the grafted plants. Also, reduction in the transcript levels of *MrPIN1* accompanied by high levels of auxin are known for stimulating the founder cells to undergo cell division, as well as cell differentiation [76]. The up-regulation of *MrPIN3* is associated with the enhanced distribution of auxins, which further induces the division of pericycle cells [77,78].

The reunion of vascular tissues is favored by the auxin movement from top to downward direction mediated by PIN proteins. One of PIN proteins, PIN1 is known to accumulate in the vascular tissue as well as in the adjacent cortex [79]. Moreover, these researchers suggested that PIN7 also regulates the development of vascular bundles (mainly in scion part) by modulating the auxin levels in the grafted plants. Accumulation of auxins at the grafting site was studied using an auxin response reporter gene (DR5::GUS, expressing GUS under the control of the DR5; a synthetic auxin response element) and it was concluded that auxins play a vital role in the reunion of vascular tissues [23]. A study on transgenic tobacco plants suggested that simultaneous expression of *iaaM* (tryptophan-2-monooxygenase, involved in biosynthesis of auxins) and *CKX* (cytokinin oxidase, involved in cytokinins degradation) in the rootstocks promotes grafting process [80]. Auxins also regulate the expression pattern of gene *HCA2* (HIGH CAMBIAL ACTIVITY2) at grafting site which is important for the reconnection of phloem [67]. Analysis of grafted *Torreya grandis* plants using transcriptome approach suggested that auxins regulate the key MAPK signaling pathway during graft development accompanied by modulation of nitrogen metabolism [72]. Moreover, auxins trigger the graft development by regulating the key metabolic pathways like phenylpropanoid, cytochrome P450 metabolism and carbohydrate metabolism [72].

There are proteins like AUXIN1 (AUX1) and LAX1-3 (LIKE AUXIN RESISTANT1-3) belonging to AUX/LAX family which encodes many proteins having same properties to that of amino acid transporters [81,82]. The expression pattern in majority of LAX genes during the graft development is down regulated [71]. However, auxin treatment is able to up-regulate the expression of *LAX1* and *LAX3* but at the same time *AUXI* and *LAX2* expressions are not affected by the application of auxin. *LAX2* is also involved in the transport of auxins during vascular development [83]. In *Carya cathayensis*, expression of AUX/IAA family genes like *CcIAAx, CcIAA8a*, *CcIAA11, CcIAA27a2*, *CcIAA27b,* and *CcIAA28* during the early stages of grafting indicated their positive role in the process by regulating the vascular connection at grafting site [84].

**Figure 1:**
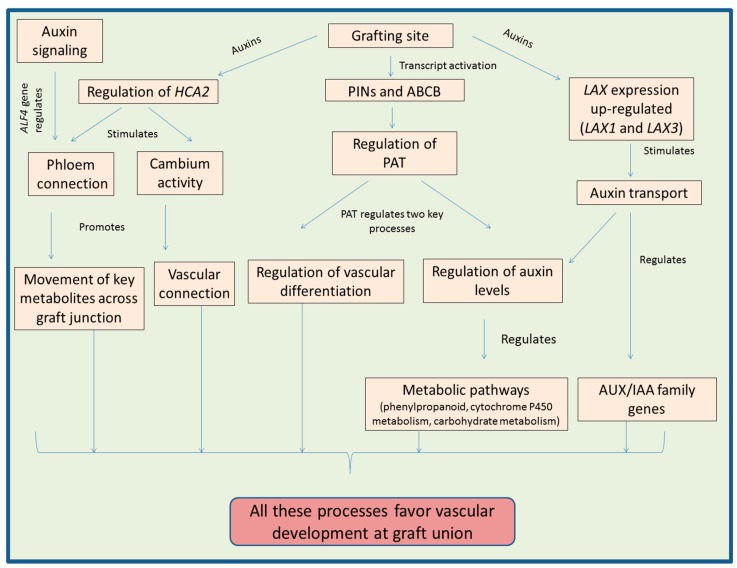
Diagram explaining how auxins regulate the process of plant grafting. It is a conclusion diagram, based on the various studies mentioned in the section related to auxins. PIN-FORMED auxin transport proteins (PINs); ATP-binding cassette subfamily B (ABCB); polar auxin transport (PAT); cytokinin (CK); gibberellins (GA); graft union (GU); LIKE AUXIN RESISTANT1-3 (LAX); HIGH CAMBIAL ACTIVITY2 (HCA2); ABERRANT LATERAL ROOT FORMATION 4 (ALF4).

Auxins are involved in the formation of lateral roots, regulation of the xylem development and cambium growth in plants [85,86,87]. Reduced auxin response in *dgt* (*Diageotropica*) *Lycopersicon esculentum* mutants resulted in the hindrance to form root primodia (lateral) accompanied by enhanced production of undifferentiated mass of cells [88,89]. Transcriptomic studies carried out by Spiegelman et al. [49] proposed that products of some genes like *S1Cyp1* function as signaling molecule which regulates the auxin response via reducing the transcription of some important genes belonging to NAC family of transcription factors. Moreover, this family has many other transcription factors which are involved in the regulation of auxin response, xylem and lateral root development, which are important factors responsible for the success of a plant graft [90,91,92]. The reconnection of phloem tissue is also controlled by genes of auxin-signaling pathway like *ABERRANT LATERAL ROOT FORMATION 4 (ALF4)* [19]. This gene (*ALF4*) plays key role in the callus as well as lateral root formation [93,94,95]. Lateral root formation is also regulated by other genes of auxin-signaling pathway including *IAA18* and *IAA28* [96]. Figure 1 provides overview of various processes regulated by auxins at graft junction. 

### 3.2. Regulation of the Grafting by Cytokinins

Cytokinins regulate plant growth and development by modulating key physiological and molecular processes [97,98,99]. This phytohormone controls processes like cell division, growth of shoot apical meristem, development of vascular system, root growth, tissue patterning and shoot organogenesis [98,99,100,101]. In addition to normal conditions, CKs also regulate plant biology under adverse environmental conditions [100,102]. Cytokinins play an important role in the growth and development of graft union by stimulating the callus proliferation at the site of tissue unions [103]. CKs biosynthesis is triggered in plants during the process of wound healing [104], suggesting their positive role in the graft development. Enhanced levels of zeatin riboside at the graft unions also favor the role of CKs during grafting [105]. This phytohormone triggers the healing of stem wounds by stimulating the regeneration process of vessels as well as sieve tubes, and CKs along with auxins promote the vascular differentiation and increase the phloem/xylem ratio [106]. It has been noticed that high levels of CKs in the xylem of rootstocks favor the auxin transport from its own shoot parts, and this enhanced auxin transport positively favors the development of graft unions [107]. Treatment of grafts with CKs results in fast graft growth and high success rate due to the enhanced callus formation, quick phloem regeneration, and increased nutrient transport to the scions from soil via stocks [108,109]. In addition to graft success, exogenous application of CKs induces the bud formation on scions after successful graft union [110]. In addition to the involvement of CKs in regulating the pattern of vascular bundles, they regulate the other key processes like root meristematic activity and root architecture [111,112]. Grafting studies carried out using wild type and CK-biosynthetic mutants showed that CKs are capable of long distance transport [113]. This transport of CKs may play an important role in the modulation of polar auxin transport accompanied by regulation of root vascular development [114,115]. Moreover, shoot biosynthesized CKs can regulate the expression of PIN proteins along with auxin signaling [115], which is important for the growth and development of grafted plants.

#### Cytokinins Regulate Cambium Activity and Secondary Growth to Favor Grafting

Cambium development is considered to play a major role during the process of grafting [116]. Cambium also undergoes differentiation in plants after cutting to healing of grafting site [19]. Cambium is highly active above the cut site and is involved in the development of vascular tissues [117]. Moreover, absence of cambium is known to restrict the plant grafting process [118]. Additionally, in incompatible grafts, the cambium formation is observed to be delayed [119]. In grafting experiments carried out on *Carya illinoensis*, cambium proliferation was noticed to play an important role in successful grafting [120]. Involvement of cambium tissue in wound healing and successful graft unions is mentioned in a literature review by Melnyk [121]. Additionally, recent studies by Melnyk et al. [67] concluded that at grafting site, genes involved in vascular development (cambium, phloem and xylem) were activated immediately after grafting. They further observed that these transcriptional changes disappeared after successful reconnection of vascular vessels and healing of the graft site. The above mentioned studies suggest that cambium plays a central role in graft development, so in upcoming text we will discuss how CKs regulate the growth and development of cambial tissue. 

In addition to their role in the division of general plant cells, cytokinins are involved in regulating the activity of vascular cells and the application of CKs along with auxins is known to trigger the division of cambium cells [122]. CKs play role in the vascular cell growth during the primary as well as secondary development of vascular bundles. Moreover, CK homeostasis also positively regulates the growth and development of cambium tissue [123,124,125]. Expression of some key genes (in vascular tissues) involved in CK biosynthesis and transport further supports the fact that CKs play an important role in regulating the vascular tissue development [126,127]. Moreover, CKs alone cannot fully regulate the growth and development of vascular tissues, but along with other hormones like auxins, they regulate cell division, xylem fiber development, and cambium activity, and enhance regeneration power of phloem/xylem tissues at the site of injury [128]. In *Populus* mutants formed after overexpression of *AtCKX2* gene (encoding a CK degrading enzyme in *Arabidopsis*), decline in the CK-signaling in cambium cells accompanied by reduction of endogenous CK levels, as well as responsiveness to CKs. All these observations were accompanied by the reduction of radial growth of stems, which might be due to the negative impact of low CK concentration on vascular cambial cell division. It supports the fact that CKs have important role in the normal functioning of vascular cambium [125]. Study on *Arabidopsis* mutants supported the fact that CKs are involved in the regulation of cambial activity. In *Arabidopsis* mutant plants (*atipt1 3 5 7*), enzymes involved in CK-biosynthesis (ATP/ADP isopentenyltransferases) were absent. This resulted in declined CK levels accompanied by reduction in cambium activity, stem diameter and length of stem in mutant plants. Further, CKs were observed to regulate the normal cambium activity in shoot and root, after grafting of a normal stock and scion respectively [113,129].

Cytokinin-signaling plays a critical role in the proliferation and development of procambium as well as cambium cells, and this fact somehow cleared doubts related to the maintaining of stem cells present in lateral meristems [123,130,131]. CK-signaling activates type-B ARR transcription factors which play critical role in the cell division and callus formation [132,133,134]. CK-activated ARR2 is known to provide immunity to plants against biotic factors via salicylic acid (SA) mediated cell signaling [135]. Recent study on *Arabidopsis* suggested that ARR11 negatively regulates SA-JA crosstalk to confer biotic immunity to plants [136]. Type-B ARRs are main regulators of CK-mediated proliferation of callus at graft junction [105]. Moreover, enhanced expression of the CK-receptor genes like *PtHK3a*, *PtHK3b* (popular), and *BpCRE1* (birch) in the cambium region suggests the positive role of CK in vascular tissue development [125]. The expression of *CRE1*/*WOL*/*AHK4* (*CYTOKININ RESPONSE 1/WOODEN LEG/*ARABIDOPSIS HISTIDINE KINASE 4) in the root vascular cylinders favors the fact that CK-signaling is involved in the proliferation as well as specification of vascular cells [137]. In grafted hickory plants, during early developmental stage, many genes involved in CK-signaling like AHK/CRE genes were up-regulated at the site of graft union (comp113371_c0, comp75037_c0, comp48382_c0 and comp90336_c0). Additionally, some type-A ARR (comp63651_c0 and comp89423_c1) and type-B ARR (comp212565_c0 and comp92083_c0) genes were also up-regulated at union point. This was accompanied by down-regulation of some genes (comp69732_c0, comp75680_c0) and ultimately, CK-signaling after activation enhanced the success rate of grafts [138]. Additionally, ARR5 expression study has suggested that CK-signaling (without any exogenous CK stimulus) gets activated in the root meristematic tissues (particularly in the intervening procambium region) [115]. Stimulated expression of *B-ARR* genes in grafted *Torreya grandis* plants also indicated the role of CK-signaling in the graft development [72].

**Figure 2:**
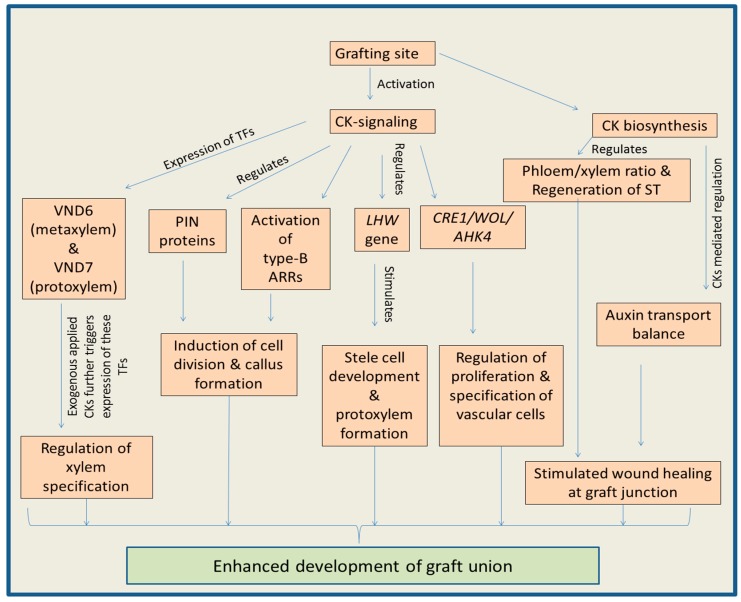
Diagram explaining how cytokinins regulate the process of plant grafting. It is a conclusion diagram, based on the various studies mentioned in the section related to cytokinins. Cytokinins (CKs); tracheary elements (TE); sieve tube (ST); PIN-FORMED auxin transport proteins (PIN); type-B Arabidopsis response regulators (type-b ARRs); VASCULAR-RELATED NAC-DOMAIN7 (VND); *CYTOKININ RESPONSE 1/WOODEN LEG/*ARABIDOPSIS HISTIDINE KINASE 4 (*CRE1*/*WOL*/*AHK4*).

Cytokinin-signaling also regulates the activity of gene *LHW* (LONESOME HIGHWAY). This gene has an important role in growth and development of stele cells as well as in formation of protoxylem [137,139]. Specification of xylem is mainly regulated by the two transcription factors named VND6 and VND7 (VASCULAR-RELATED NAC-DOMAIN). It has been observed that VND6 expresses during metaxylem formation, whereas VND7 expresses during protoxylem formation. The expression of these two key transcription factors enhances significantly after CK application, indicating the role of CKs in xylem development [137,140]. This phytohormone controls the differentiation of tracheary elements by posttranscriptional regulation of xylogen transcripts leading to the accumulation of xylogen protein [137,141]. Declined levels of CKs and reduced CK cell signaling in the cambium cells of *Populus* trees (BpCRE1::AtCKX2, in which *Arabidopsis CYTOKININ OXIDASE 2* gene is expressed under promoter of *Betula pendula CYTOKININ RECEPTOR 1* gene) were accompanied by the decreased division of periclinal cells, which indicated involvement CKs in vascular tissue development [125]. It has been noticed that CK mediated regulation of the shoot vascular tissue formation is dependent upon the working of key signaling receptors like AHK2, AHK3 and CKI1 His kinase (CYTOKININ INDEPENDENT 1) [124,142,143]. CKI1 expresses in the stem vascular tissues. Moreover, in mutants (*ahk2* and *ahk3*), the function loss lead to decline in the CK concentration accompanied by hampered development of procambium and secondary growth. However, overexpression of CKI1 resulted in recovery of vascular tissues in *ahk2* and *ahk3* mutants, suggesting that CK-regulates AHK2 and AHK3 [144]. Genes involved in CK-signaling (including family of CK receptor genes) are known to express in the cambium of plants like *Populus trichocarpa* and *Betula pendula*. However, the overexpression of *CKX* gene (encoding enzymes cytokinin oxidase/dehydrogenase, responsible for degradation of CKs) in the cambium region causes reduction in the stem diameter, indicating a possible function of CKs in the development of vascular tissues [125,145]. The detailed explanation of the various functions of CKs during the grafting is shown in Figure 2.

### 3.3. Regulation of Grafting by Gibberellins

The gibberellins (GAs) are an important class of phytohormones well known for their roles in regulation of plant growth and development [146,147]. They regulate key biological processes like cell division, cell elongation, late embryogenesis, and delay in fruit ripening [148,149,150]. Plant biological processes are also regulated by GAs under abiotic stress conditions via GA-signaling pathways [151]. GAs are key regulators of the plant vascular growth and are known to modulate the processes like cambium activity, xylem fiber differentiation, xylem expansion and plant secondary growth [147,152]. High success rate of grafts was observed in *Syzigium cuminii* under red light and it was suggested that this was mediated by GAs, as the conversion of inactive GAs to the active form is promoted by far-red light [153]. GAs are also capable of moving across the graft union [152,154]. This GA movement is suggested as key factor in the joining of scion-stock, and normal vascular development of plants [152,155]. All these processes are important for the success of grafts, indicating the positive role of GAs in grafting. Involvement of GAs in the reunion of cortex in cucumber and tomato further favors the role of this phytohormone during graft unions [156,157,158]. GAs boost the process of xylogenesis, supporting the fact that GAs have the potential to trigger the formation of vascular bundles at the site of graft unions [105,159]. In plant stems, GAs regulate cambium activity as well as xylem fiber differentiation. The studies carried out by Dayan et al. [147] showed that defoliated tobacco plants had lower concentrations of endogenous GAs followed by significant reduction in the activity of cambium and declined xylem fiber differentiation. However, in normal plants, the vascular activity and secondary plant growth were found to be normal along with normal GA concentration. GA-mediated regulation of vascular bundle fibers was observed after studying the expression pattern of GA-inducible promoters of genes like EXP1, MYB34 and GA2OX2 [147]. Over expression of GA biosynthetic genes, or silencing of genes responsible for deactivating GA biosynthetic pathway are observed to boost the xylem tissue development and overall stem growth [160,161,162].

In hypocotyl, GAs regulate the expansion of xylem tissue. This fact was supported by studying the *gal-3* mutants (in which biosynthesis of GAs was blocked). Results suggested that the xylem tissue development in *gal-3* mutants was impaired, but grafting of normal scion over mutant rootstocks resulted in the recovery of xylem tissue expansion [152]. Grafting experiments between mutants (lacking GA-biosynthetic enzymes) and wild type pea plants suggested the role of GAs in regulating the normal plant growth [163]. GAs along with their signaling pathways are also known to induce and boost the process of wood formation. Moreover, GA-signaling is involved in the regulation of xylem expansion. This fact was supported by the study in which GA-receptor, GID1a was mutated. Mutant *gid1a* showed absence of xylem expansion, indicating the role of GA-receptors and signaling [152]. GA-signaling pathway interacts with auxin pathway to regulate the activity of cambium in plants [164]. Ratio of auxins and GAs determines the effect on vascular tissues. Higher ratios of IAA:GA induce the formation of xylem, whereas low ratios of IAA:GA induce the formation of phloem [147]. In combination, both these phytohormones (auxin and GAs) regulate the cell division as well as secondary growth of vascular tissues. In the cambial region, GAs are known to stimulate PAT by up-regulating key auxin transporter PIN1 [164,165].

**Figure 3:**
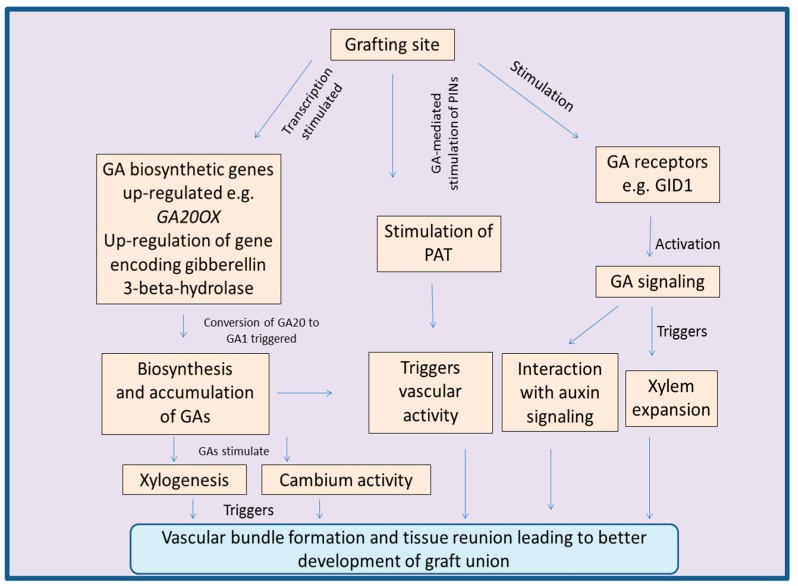
Diagram explaining how gibberellins regulate the process of plant grafting. It is a conclusion diagram, based on the various studies mentioned in the section related to gibberellins. Gibberellins (GA); graft union (GU); PIN-FORMED auxin transport proteins (PINs); polar auxin transport (PAT); GIBBERELLIN INSENSITIVE DWARF1 (GID1); Gibberellin 20-oxidase (*GA20OX*).

It was noticed that wound healing or tissue reunion process in cucumber hypocotyls was enhanced after the application of GA to cotyledonless plants. Moreover, the application of uniconazole-P (an inhibitor of GA-biosynthetic pathway) to the cotyledons resulted in declined cell division of cortical tissues at the site of junction [157,166]. Additionally, endogenous levels of GAs were found to be higher in seedlings with cotyledons, as compared to that of seedlings without cotyledons. This was accompanied by successful tissue reunion after incision in seedlings with cotyledons, whereas tissue reunion was poor in the cotyledonless seedlings [158]. The reason behind this is GA-mediated division and elongation of cortical cells, which are involved in the connection of stems [166]. Exogenous application of GAs to the apple rootstocks also promotes the length and number of nodes [167]. At the site of graft union, concentration of GAs is known to enhance along with up-regulation of the expression of *GA20OX*, a gene involved in GA-biosynthesis. Additionally, the expression of genes involved in the deactivation of GA-biosynthetic pathway, was down-regulated [105]. After grafting in watermelon, regulation of a gene encoding gibberellin 3-beta-hydroxylase (*Cla015407*), a key enzyme involved in the conversion of GA20 to GA1, also suggests role of GAs during the process of grafting [168]. Grafting scion of transgenic *Jatropha curcas* with over expressed gene *JcGA20ox1* (encoding gibberellin 20-oxidase, a GA-biosynthetic enzyme) upon the wild type rootstocks resulted in successful graft. The resulting plants had enhanced stem elongation accompanied by increased outgrowth of lateral buds. The stimulatory role of GAs mentioned above was further confirmed by reduced lateral bud growth after application of paclobutrazol, an inhibitor of the GA-biosynthetic pathway [169]. Figure 3 concludes the various process regulated by GAs during the process of grafting.

## 4. Hormonal Crosstalk at Grafting Site

**Figure 4:**
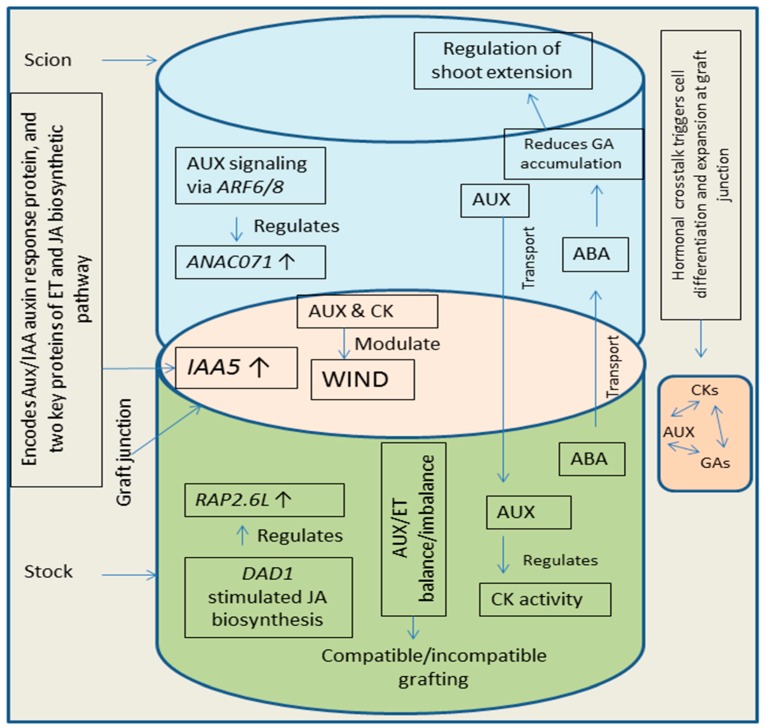
Diagram showing hormonal crosstalk during the process of grafting. It is a conclusion diagram, based on the various studies mentioned in the section related to hormonal crosstalk. *IAA5* (*INDOLE-3-ACETIC ACID INDUCIBLE 5*); AUX (auxins); CK (cytokinin); ET (ethylene); JA (jasmonic acid); ABA (abscisic acid); *ANAC071 (NAC DOMAIN CONTAINING PROTEIN 71); RAP2.6L (RELATED TO AP2 6L); ARF (AUXIN RESPONSE FACTOR); DAD1 (DEFECTIVE ANTHER DEHISCENCE 1);* *↑* (up-regulation).

Plant grafting is controlled by hormones and crosstalk among different hormones play a key role in the development of graft unions [22,24]. The vascular reconnection in grafted plants involves the role of many plant hormones like auxins, CKs, and GAs [1,57]. Wound induced differentiation 1 (WIND1, a key transcription factor induced by wounding and is involved in plant cell dedifferentiation) plays role in the process of cell dedifferentiation taking place during wound healing at graft union [170]. Plant hormones like auxins and CKs modulate WIND1 pathway, leading to the regulation of vascular reunion process [24,170]. Additionally, ET and JA are also involved in the regulation of vascular reunion process during plant grafting [23,171]. Formation of callus at the site of graft is initiated by WIND1 mediated CK response [170]. Crosstalk between auxins and CKs has key functions in the connection of vascular tissues [172]. Auxins are known to regulate the biosynthesis and activity of CKs (synthesis in roots of stock and translocated to scion part) leading to modulation of shoot growth [173]. After incision, soon the expression of IAA5 (INDOLE-3-ACETIC ACID INDUCIBLE 5) gets enhanced. IAA5 encodes an Aux/IAA auxin response protein, and two key proteins of ET and JA biosynthetic pathway (1-AMINO-CYCLOPROPANE-1-CARBOXYLATE SYNTHASE 2 and LIPOXYGENASE 2) [166] suggesting a possible crosstalk among auxins, ET and JA at graft junction. Simultaneously, accumulation of auxin increases above the site of grafting and decreases in below grafting part. This enhanced auxin concentration along with ET mediated signaling, enhances the expression of *NAC DOMAIN CONTAINING PROTEIN 71 (ANAC071*) accompanied by reduction in the expression of *RELATED TO AP2 6L* (*RAP2.6L*) and JA levels [24,166]. Moreover, JA levels and expression of *RAP2.6L* get recovered due to the less auxin concentration in the below grafting parts. The enhanced expression of *ANAC071* (due to auxin signaling via ARF6/8, above grafting site) and *RAP2.6L* (due to JA biosynthesis via induction of DEFECTIVE ANTHER DEHISCENCE 1, below grafting site) favors the process of vascular reunion by stimulating the cell division (vascular cells) [166,174]. Additionally, crosstalk of auxins with CKs and GAs increases the pace of the vascular reunion by triggering cell differentiation and cell expansion [24]. Abscisic acid (ABA) movement take place from stock to scion part [175] and is known to regulate shoot extension by reducing the GA1 accumulation [176] which suggests a possible crosstalk between ABA and GA, needing further detailed studies. Grafting experiments (in grapevine) revealed hormonal regulation (auxins, CKs, ET, JA) of graft formation [177] indicating that all these phytohormones crosstalk to control graft development. Additionally, recent studies on apple grafting concluded that interaction of exogenously applied CKs and GAs regulates the branching pattern in grafted plants [178]. Scion growth gets triggered due to enhancement and reduction in the supply of CKs and auxins respectively, into shoot part [179]. Balance between auxins and ET is important for successful grafting and imbalance between these hormones can cause inhibition in grafting process [180]. Enhanced over accumulation of auxins in the roots (basipetally-transported) promotes the accumulation of ET in roots, which further favors production of reactive oxygen species leading to reduction of root growth and failure of grafts [22,180]. Figure 4 provides an overview of hormonal crosstalk in plants at grafting site.

## 5. Conclusions and Future Prospects

During grafting, various physiological and molecular processes take place at connection site, which are responsible for the tissue union. Researchers all over the world are interested in studying the deep mechanisms behind grafting process, so that implications based on available information can be applied to improve the success rate of plant grafts. Plant hormones are actively involved in regulation of grafting process and their role during the process of graft development is important to study. After reviewing the latest developments in the field, it is concluded that application of molecular techniques in horticultural field along with the application of phytohormones can be helpful in the production of improved crop varieties.
